# Study on new creep equation using discrete cosine transform for high temperature materials

**DOI:** 10.1016/j.heliyon.2019.e02619

**Published:** 2019-11-01

**Authors:** Hideo Hiraguchi

**Affiliations:** The Institution of Professional Engineers, Japan, 5-8, 3-Chome, Shiba-koen, Minato-ku, Tokyo, 105-0011, Japan

**Keywords:** Materials science, Modified θ projection, Fourier transform, Discrete cosine transform, Creep strain rate

## Abstract

For an equation representing creep strain-time relationship curves of high temperature materials, the equations which represent from the primary creep region to the tertiary creep region using exponential functions such as the modified θ projection [3] have already been proposed. However, the estimated values by the modified θ projection don't necessarily coincide with the measured values, and deviations occur between the estimated values and the measured values [8]. Then in this paper, we propose a new creep equation using the Discrete Cosine Transform (DCT). According to this equation, all the estimated values coincide with the measured values and each interpolated value between the measured points gives a reasonable value. Therefore it is possible to express a creep curve quite well from the primary creep region to the tertiary creep region by using the DCT.

## 1 Introduction

Equations using power law such as Bailey-Norton method [1, 2] (Eq. 1) and exponential functions like the modified θ projection [3] (Eq. 2) have already been proposed as equations representing creep strain-time relationship curves of high temperature materials for such as boilers and turbines.

(1) *ε* = *ε*_0_ + *Aσ*^*n*^*ｔ*^*m*^

(2) *ε* = *ε*_0_ + *A*1･{1 –exp (-*αt*)} + *B*1･{exp(*αt*) - 1}

where, *ε*: total strain, *ε*_0_: initial strain, *σ*: stress, *t*: time, *A*, *n*, *m*, *A*1, *α*, *B*1 are material constants.

The modified θ projection expressed in Eq. (2) is simplified by decreasing by one constant from the material constants of the θ projection of Evans and Wilshire [4] derived from the fact that creep deformation is made by two processes of work hardening and softening. Moreover, it is known to display the entire creep curve from the primary creep region to the tertiary creep region.

In this paper, we propose an unprecedented new equation using the discrete cosine transform that can express from the primary creep region to the tertiary creep region instead of the above Eqs. (1) and (2).

The content of this research is a slightly modified version of what was presented at the 56th Symposium on Strength of Materials at High Temperatures of the Society of Materials Science, Japan in 2018 [9].

## 2 Materials and methods

### 2.1 Ni based superalloy

For this study, Nickel based 16Cr-8.5Co-3.5Al-3.5Ti-2.6W-1.8Mo-0.9Nb superalloy from the database (No.49A) of National Institute for Materials Science (NIMS) was selected. Since this superalloy is used for materials at high temperature and high pressure such as gas turbine components or so, investigating the creep phenomena of the superalloy by using the discrete cosine transform (DCT) will be able to contribute to the advancement of new materials analysis technology.

### 2.2 About discrete cosine transform

The discrete cosine transform (DCT) is a kind of Fourier transform that can be processed only with a cosine component which is a real number [5], since the imaginary term of the Fourier transform becomes zero when the original data is an even function. The discrete Fourier transform is expressed as follows;

(3) $$X\text{[}k\text{] }=\;\overset{N-1}{\underset{n=0}{\sum }}x[n\text{] exp(}-2\pi \mathrm{nkj}\text{ /}N\text{), (}k=\text{0,}1\text{,}\cdot \cdot \cdot \text{, }N-1\text{)}$$

where, *x*[*n*] is a discrete signal, *N* is the number of data. If *x*[*n*] is an even function, the imaginary term is zero. Therefore, the DCT equation can be expressed as follows [5];

(4) $$X\text{[}k\text{] }=\;\overset{N-1}{\underset{n=0}{\sum }}x[n\text{] cos(}2\pi nk\text{ /}N\text{)}$$

Moreover, type II DCT of Eq. (5–1), (5–2), (5–3) used for image processing such as JPEG and MPEG was used as the DCT equation [5, 6, 7].

(5–1) $$X\text{[}k\text{] }=\;\sqrt{\text{(}2\text{/}N\text{)}}\cdot \text{c[}k\text{]}\overset{N-1}{\underset{n=0}{\sum }}x[n\text{] cos\{(}2n+1\text{)}k\pi /2N\text{\}}$$

(5–2) $$x\text{[}n\text{] }=\;\sqrt{\text{(}2\text{/}N\text{)}}\cdot \sum_{k=0}^{N-1}\text{c[}k\text{]}\;\cdot \text{X[}K\text{]}\cdot \text{cos\{(}2n+1\text{)}k\pi /2N\text{\}}$$

(5–3) $$c\text{[}k\text{] }=\;1\text{/}\sqrt{2}\text{, }k\;=\text{ 0}\;1\;\text{, k }\neq \text{ 0}$$

### 2.3 Application to creep equation

The creep data used for the discrete cosine transform must be obtained by measuring the creep strain at the same measurement time interval. However, since the measurement time of the data of National Institute for Materials Science (NIMS) used for the analysis in this study is not equally spaced, we approximated the measured points by the modified θ projection and calculated equidistant creep strain-time data from this approximated equation.

For example, the creep curve of 750 °C and 500 MPa of the Ni based superalloy of NIMS (No.49A) was approximated by the modified θ projection, and creep data of 8 points were created at 80 h intervals. These eight equally-spaced data as *x*[*n*] (*n* = 0 to 7) were DCT-transformed to *X*[*k*] according to Eq. (5-1). Then values of the discrete cosine series (inverse discrete cosine transform (IDCT)) were calculated from Eq. (5-2) using the obtained *X*[*k*] (k = 0 to 7).

**Fig. 1:**
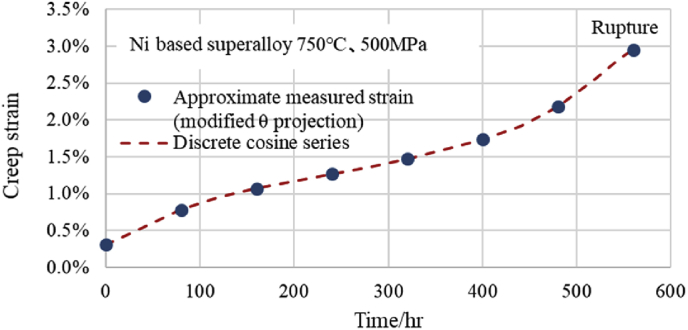
Fitting situation between measured strains and DCT-values.

**Fig. 2:**
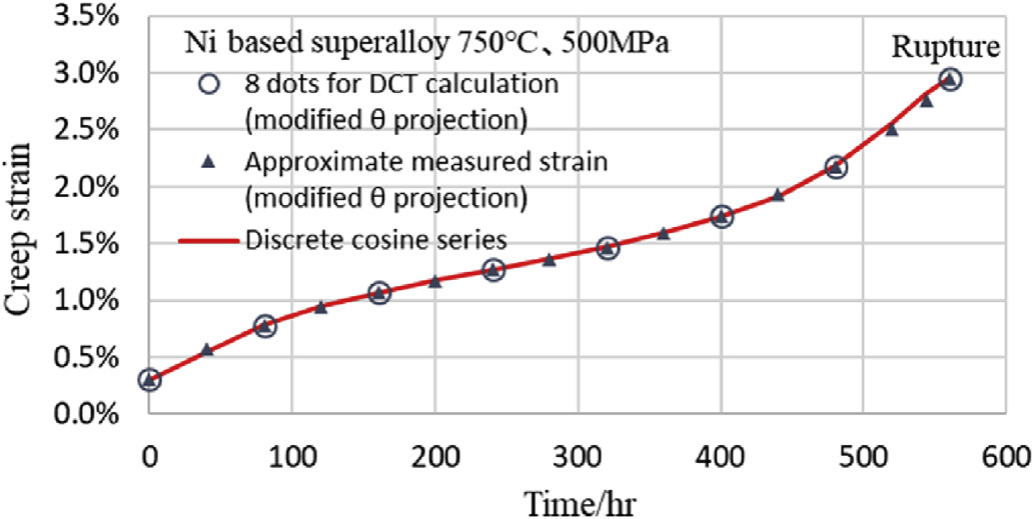
Creep curve fitted with discrete cosine series by DCT. Comparison between 16 dots and discrete cosine series.

Although the number of obtained *X*[*k*] is 8, it is very easy to perform DCT transform to obtain the result of Eq. (5-2) on a computer. Further, it is possible to easily use Eq. (5-2) as a creep equation having eight *X*[*k*]. By inputting the eight values obtained as *X*[*k*] from Eq. (5-1) into Eq. (5-2) as coefficients, creep strain can be calculated using *k* and *n* obtained by dividing the measurement time by the interval as a variable. The fitting situation of this discrete cosine series is shown in Fig. 1. It can be seen that the discrete cosine series passes through all 8 approximate measured points from the primary creep region to the tertiary creep region. In the author's previous paper, a similar result was obtained at Ni based superalloy at 750 °C and 550 MPa [8]. In addition, Fig. 2 shows the comparison situation of a total of 16 estimated points obtained by adding intermediate points (3/4 point for 16th point) to the 8 points in Fig. 1 and the estimated values from the discrete cosine series. As shown in Fig. 2, at two points on the left side of the rupture point, minute deviations are observed between the estimated creep strain using the modified θ projection and DCT points, but the other points are consistent with each other. However, the two points of the discrete cosine series just before rupture are being shifted to the safe side.

**Fig. 3:**
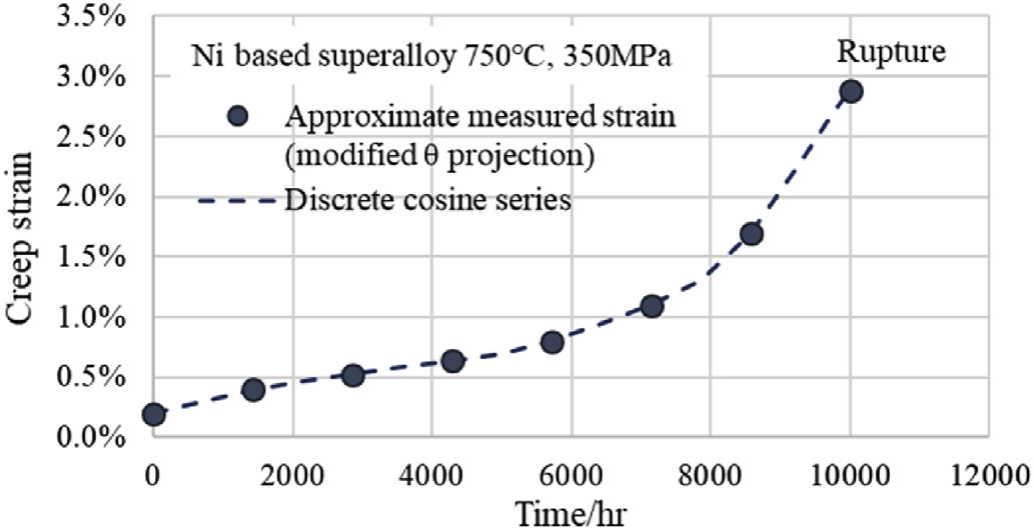
Fitting situation between measured strains and DCT-values.

**Fig. 4:**
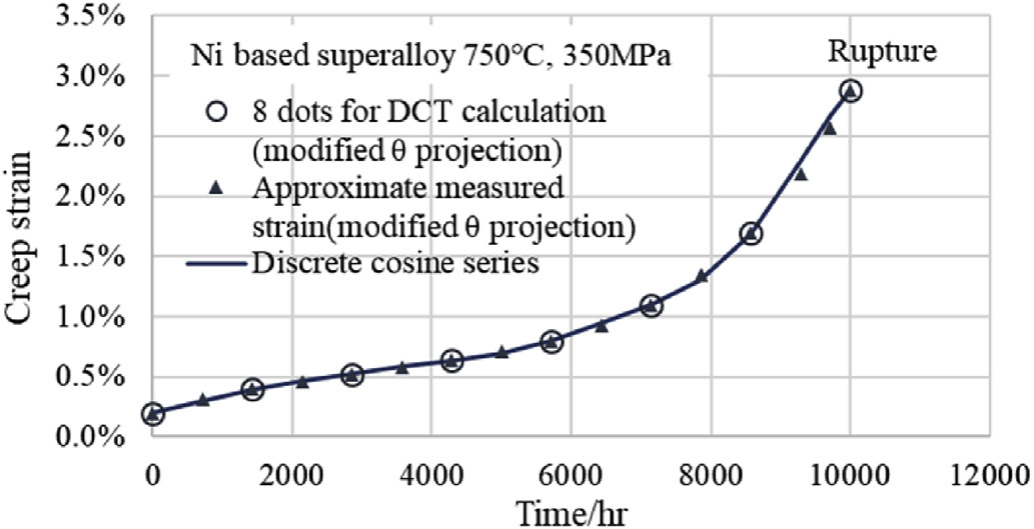
Creep curve fitted with discrete cosine series by DCT. Comparison between 16 dots and discrete cosine series.

Furthermore, to support the above results, the creep curve of 750 °C and 350 MPa of the Ni based superalloy of NIMS (No.49A) was approximated by the modified θ projection, and creep data of 8 points were created at 1,430 h intervals for DCT. Then the DCT was performed in the same way as the above and the DCT curve of 750 °C and 350 MPa was obtained. The fitting situation of this discrete cosine series is shown in Fig. 3. It can be seen that the discrete cosine series passes through all 8 approximate measured points from the primary creep region to the tertiary creep region as well as Fig. 1. Moreover, Fig. 4 shows the comparison situation of a total of 16 estimated points obtained by adding intermediate points (3/4 point for 16th point) to the 8 points in Fig. 3 and the estimated values from the discrete cosine series. As shown in Fig. 4, at two points on the left side of the rupture point, minute deviations are observed between the estimated creep strain using the modified θ projection and DCT points, but the other points are consistent with each other as well as Fig. 2. However, the two points of the discrete cosine series just before rupture are being shifted to the safe side as well as Fig. 2.

These results from Figs. 2 and 4 indicate that it is possible to estimate by interpolation if there are 8 measured values at equal time intervals.

### 2.4 Calculation and consideration of creep strain rate

We calculated the creep strain rate obtained from the time derivative of Eq. (5-2) of the discrete cosine series. As a result, an equation represented by following Eq. (6) was obtained.

(6) $$\text{d}x\text{[}n\text{]/d}t\;=\;-\text{ (}N-1\text{) / }t_{\mathrm{total}}\cdot \sqrt{\text{(}2\text{/}N^{3}\text{)}}\cdot \pi \overset{N-1}{\underset{k=0}{\sum }}k\cdot \;c\text{[}k\text{]}\cdot X\text{[}k\text{]}\cdot \text{sin((}2n+1\text{)}k\pi /2N\text{)}$$

where *N* is the number of data, *t* is the measurement time, *t*_*total*_ is the total measurement time.

**Fig. 5:**
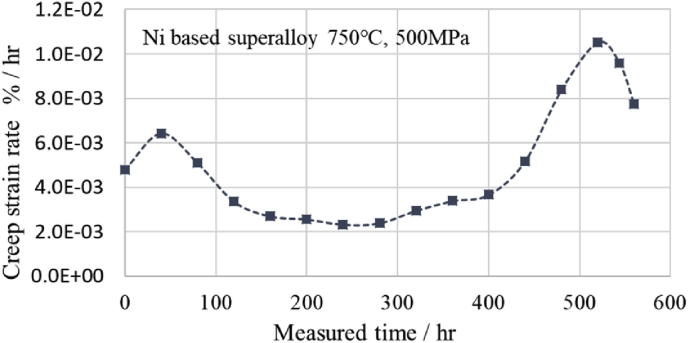
Creep strain rate derived from first derivative of discrete cosine series.

**Fig. 6:**
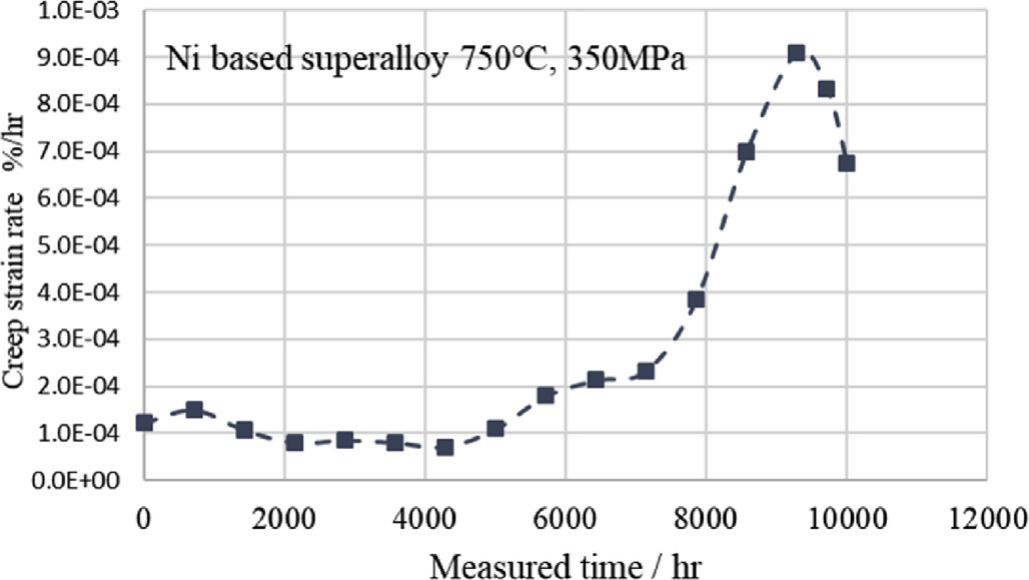
Creep strain rate derived from first derivative of discrete cosine series.

Fig. 5 shows the estimated creep strain rate curves derived from Eq. (6) by DCT using 8 points data obtained by the modified θ projection of the Ni-based superalloy of Fig. 2 at 750 °C and 500 MPa Fig. 6 shows the estimated creep strain rate curves derived from Eq. (6) by DCT using 8 points data obtained by the modified θ projection of the Ni-based superalloy of Fig. 4 at 750 °C and 350 MPa. In Fig. 5, when the measurement time is 240 h, the minimum creep strain rate is 2.33E^−3^ (%/hr), which is somewhat smaller than the minimum creep strain rate of 3.35 E^−3^ (%/hr) of NIMS (No.49A), but is a reasonable value. In Fig. 6, when the measurement time is 4,300 h, the minimum creep strain rate is 7.21 E^−5^ (%/hr), which is also somewhat smaller than the minimum creep strain rate of 9.86 E^−5^ (%/hr) of NIMS (No.49A), but is a reasonable value too. Therefore, it is considered that Eq. (6) can be useful to calculate the creep strain.

### 2.5 Stress dependence of discrete cosine transform

**Fig. 7:**
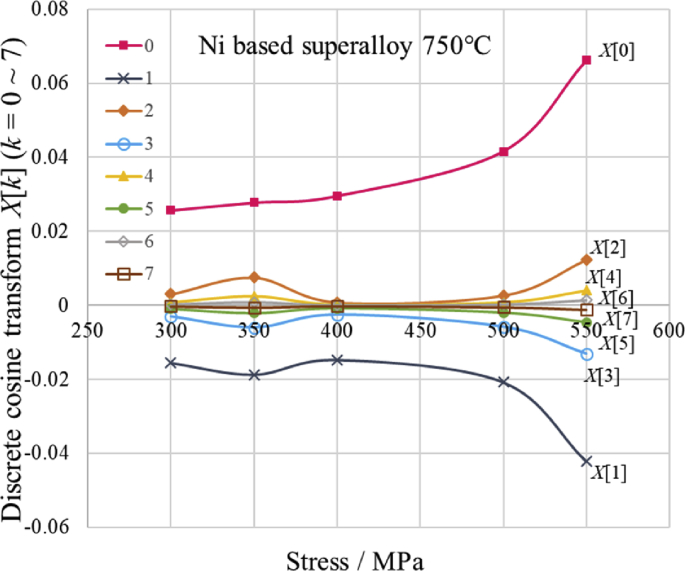
Relationship between Discrete cosine Tranform *X*[*K*] (*k =* 0∼7) and stress.

Figs. 1, 2, 3, 4, 5, and 6 are the DCT analysis results of Ni based superalloy at 750 °C and 500 MPa or 350 MPa. Then in order to confirm the stress dependence of *X* [*k* ], *X* [*k* ] (*k* = 0 to 7) when pressure of each DCT case is 300, 350, 400, 500 and 550 MPa are shown in Fig. 7. In Fig. 7, it can be seen that there are two types, which increases and decreases as increasing stress, except for 350 MPa. When the pressure is 350 MPa, it is considered that peaks and valleys have occurred in multiple curves due to some change in the metallographic structure. By examining the change of the discrete cosine transform *X* [*k* ] (*k* = 0 to 7) accompanying the stress change, it can be expected that the metallographic change can be grasped.

## 3 Discussion and conclusion

From the results of this research and the author's previous published papers [8, 9], it was found that the discrete cosine transform and series, which is an improved version of the Fourier transform, is suitable for the creep equation and the creep strain rate equation. The summary is as follows;

1) The discrete cosine transform and series fits well to the relationship curve between the creep strain and time from the primary creep region to the tertiary creep region (Figs. 1, 2, 3, and 4). In addition, it is possible to estimate creep strains by interpolation if there are 8 measured values at equal time intervals (Figs. 2 and 4).
2) The first derivative of the discrete cosine series with respect to time gives a reasonable value for the creep strain rate at each measurement time (Figs. 5 and 6. Therefore, it is considered that Eq. (6) can be useful to calculate the creep strain rates including minimum creep strain rate. The minimum creep strain rates derived from Eq. (6) are somewhat lower than those of NIMS. This needs further consideration.
3) Two types of stress dependence are seen in the discrete cosine transform *X* [*k*] (*k* = 0 to 7), which increases and decreases as increasing stress, except for 350 MPa (Fig. 7). By examining the change of the discrete cosine transform *X* [*k* ] (*k* = 0 to 7) accompanying the stress change, it can be expected that the information of the metallographic change can be extracted (Fig. 7).

## Declarations

### Author contribution statement

Hideo Hiraguchi: Conceived and designed the experiments; Performed the experiments; Analyzed and interpreted the data; Contributed reagents, materials, analysis tools or data; Wrote the paper.

### Funding statement

This research did not receive any specific grant from funding agencies in the public, commercial, or not-for-profit sectors.

### Competing interest statement

The authors declare no conflict of interest.

### Additional information

No additional information is available for this paper.
